# Subclinical thyroid disorders and cognitive performance among adolescents in the United States

**DOI:** 10.1186/1471-2431-6-12

**Published:** 2006-04-19

**Authors:** Tiejian Wu, Joanne W Flowers, Fred Tudiver, Jim L Wilson, Natavut Punyasavatsut

**Affiliations:** 1 Department of Public Health, East Tennessee State University, Johnson City, TN 37614, USA; 2 Department of Family Medicine, East Tennessee State University, Johnson City, TN 37614, USA; 3 Department of Pediatrics, East Tennessee State University, Johnson City, TN 37614, USA

## Abstract

**Background:**

Thyroid hormone plays a crucial role in the growth and function of the central nervous system. The purpose of the study was to examine the relationships between the status of subclinical thyroid conditions and cognition among adolescents in the United States.

**Methods:**

Study sample included 1,327 adolescents 13 to 16 years old who participated in the Third National Health and Nutrition Examination Survey (NHANES III). Serum thyroxine (T4) and thyroid stimulating hormone (TSH) were measured and subclinical hypothyroidism, subclinical hyperthyroidism, and euthyroid groups were defined. Cognitive performance was assessed using the subscales of the Wide Range Achievement Test-Revised (WRAT-R) and the Wechsler Intelligence Scale for Children-Revised (WISC-R). The age-corrected scaled scores for arithmetic, reading, block design, and digit span were derived from the cognitive assessments.

**Results:**

Subclinical hypothyroidism was found in 1.7% and subclinical hyperthyroidism was found in 2.3% of the adolescents. Cognitive assessment scores on average tended to be lower in adolescents with subclinical hyperthyroidism and higher in those with subclinical hypothyroidism than the score for the euthyroid group. Adolescents with subclinical hypothyroidism had significantly better scores in block design and reading than the euthyroid subjects even after adjustment for a number of variables including sex, age, and family income level.

**Conclusion:**

Subclinical hypothyroidism was associated with better performance in some areas of cognitive functions while subclinical hyperthyroidism could be a potential risk factor.

## Background

Thyroid hormone plays a crucial role as a regulator of nervous system myelination, of growth and puberty, and of metabolism and organ functions [1]. It has been well recognized that hypothyroidism at early stages of life, such as congenital hypothyroidism, can cause various abnormalities including mental retardation and delayed myelination [2,3]. Disorders affecting the thyroid gland represent the most common endocrine pathology in childhood [4]. Pediatric Graves' disease, the most common cause of hyperthyroidism in children, can adversely alter growth and development including poor weight gain and hyperactivity [5].

Thyroid function disorder is a graded phenomenon. There is mounting evidence to suggest that mild (subclinical) thyroid disorders may be potential contributors to significant clinical conditions [6]. However, the relationship between subclinical hypo-hyperthyroidism and cognition among adolescents has been studied much less. In the Third National Health and Nutrition Examination Survey (NHANES III), serum total thyroxine (T4) and thyroid stimulating hormone (TSH) were measured, and cognitive function was assessed for adolescents 12 years or older. Using the data from NHANES III, this study examined the relationship of subclinical hypo-hyperthyroidism with cognitive performance among adolescents in the United States.

## Methods

### Data and study sample

Data from the Third National Health and Nutrition Examination Survey (NHANES III) were used for this study. Using a stratified multi-stage probability sampling design, NHANES III collected data representative of the non-institutionalized civilian U.S. population, aged two months or older, in the 50 states and District of Columbia, 1988–1994 (n = 39,695). Detailed information on the sample design and operation of NHANES III can be found elsewhere [7,8].

In the youth sample of NHANES III, there were 2,216 children 12 to 16 years old at the time of the interview. Among them, 1,444 (65.2%) had measurements of both serum thyroxine and thyroid stimulating hormone. Adolescents who did not participate in the cognitive assessment were excluded from this study. One subject who reported use of medication for thyroid disorder was also excluded. Consequently, the sample used in this study consists of 1,327 adolescents.

### Variables of interest

T4 was the sum of the free and protein bound forms in serum and measured by radioimmunoassay. The reference range of T4 was 4.5 to 13.2 μg/dL. Serum TSH was measured by a chemiluminescence immunometric assay utilizing a mouse monoclonal antibody to TSH immobilized on a polystyrene bead and a goat polyclonal antibody to TSH conjugated with an acridinium ester. The reference range of TSH was 0.39 to 4.6 mIU/L. The measurements of T4 and TSH were detailed in NHANES III [9] and elsewhere [10]. Hyperthyroidism was defined as clinically significant if TSH <0.1 mIU/L and T4 > 13.2 μg/dl and as subclinical when TSH <0.39 mIU/L and T4 ≤ 13.2 μg/dl. Hypothyroidism was defined as clinically significant if TSH > 10 mIU/L and T4 < 4.5 μg/dl and subclinical when TSH > 4.6 mIU/L and T4 ≥ 4.5 μg/dl.

Cognitive function was evaluated using the subsets of two tests: the Wide Range Achievement Test – Revised (WRAT-R) and the Wechsler Intelligence Scale for Children – Revised (WISC-R) [11,12]. Two subsets of the WRAT-R test, arithmetic and reading, were administered. Two subsets of the WISC-R test, a verbal component (Digit Span) and a performance exam (Block Design), were also administered. The WISC-R test was administered first and was followed by the WRAT-R test. Raw scores were recorded. Standardized scores were determined using the calculations provided in the WRAT-R and WISC-R manuals. The age-corrected scaled scores for the subsets, which are comparable between WRAT-R and WISC-R, were provided in NHANES III data set and were analyzed in this study. The cognitive assessments were performed according to a standard protocol described in the NHANES III documentation [13].

Other variables of interest included sex, race/ethnicity, age, poverty income ratio, language used, and the examiner who conducted the cognitive assessment. These variables were based on the operational definitions set forth in the NHANES III. Race/ethnicity referred to the following four categories as reported by the primary respondent in the screening and family interview portion of the survey: non-Hispanic white, non-Hispanic African American, Mexican American, and other (i.e., Hispanics not of Mexican origin or non-Hispanics from racial groups other than white or African American). Age used in this study referred to chronological age (in completed months) at the time of the NHANES III examination and was converted (divided by 12) to years for analysis. The poverty income ratio was the ratio of the reported family incomes divided by the poverty threshold, which is produced annually by the Census Bureau and adjusted for changes caused by inflation. The variable was categorized into <1, 1–2, >2, and a missing category was also created for analysis. Families were below the poverty line if their poverty income ratios were less than one. In the cognitive assessment, Spanish was used as alternative language to English. Codes for the examiners who conducted the cognitive assessment were recorded in the NHANES III data set.

### Data analysis

Descriptive statistics including proportions and means were used to describe the characteristics of the study sample. General linear models were used to compare mean levels of cognitive assessment scores among adolescents with the subclinical hyperthyroidism and with subclinical hyperthyroidism to the mean score among the euthyroid group. The cognitive assessment scores among the different groups were further analyzed with adjustment for sex, race/ethnicity, age, poverty income ratio, language used, and examiner in the cognitive assessment by including these variables in the models. To account for sampling strategy, such as over-sampling and clustering in the NHANES III, all the general linear models were performed using SPSS Complex Samples (an add-on module for SPSS for Windows) for complex sampling design with application of the weights established for the NHANES III exam sample [14].

The NHANES III data sets are accessible at the US Centers for Disease Control and Prevention's website. Because our study was a secondary analysis of the data existing in the public domain, it was exempt from review by the East Tennessee State University Institutional Review Board. The study was carried out in compliance with internationally recognized guidelines on ethics.

## Results

The study sample consisted of similar numbers of males and females. For a small number of adolescents, cognitive function was assessed in Spanish. African Americans, Mexican Americans, and adolescents from low-income families were over-represented in the sample as indicated by the percentages (Table 1), reflecting the design feature of NHANES III. A total of ten examiners performed the cognitive assessments for the study sample.

**Table 1:** Characteristics of the study sample (n = 1,327)

Characteristics	n	%
Sex:		
Boys	610	46.0
Girls	717	54.0
Ethnicity/race:		
White	346	26.1
African American	458	34.5
Mexican American	449	33.8
Other	74	5.6
Poverty Income Index:		
Not known	106	8.0
<1	429	32.3
1–2	354	26.7
>2	438	33.0
Test language:		
Spanish	52	3.9
English	1275	96.1
Age (in years):		
13	321	24.2
14	347	26.1
15	320	24.1
16	339	25.5

Twenty two subjects (1.7%) were classified as having subclinical hypothyroidism and 30 subjects (2.3%) as having subclinical hyperthyroidism. There was no clinical hyperthyroidism or clinical hypothyroidism identified by our criteria. Three subjects had TSH levels above 10 μg/dL but T4 levels all were within the reference range.

Mean scores of each cognitive assessment tended to be lower in adolescents with subclinical hyperthyroidism and higher in adolescents with subclinical hypothyroidism than that in the euthyroid group (Table 2). For example, mean scores of block design were 8.36 for the subclinical hyperthyroidism group, 9.19 for the euthyroid group, and 11.62 for the subclinical hypothyroidism. Mean scores in reading and block design were significantly higher in the adolescents with subclinical hypothyroidism than the scores in the euthyroid group.

**Table 2:** Cognitive assessment scores by thyroid condition among adolescents: The Third National Health and Nutrition Examination Survey, 1988–94

		Arithmetic		Reading		Block Design		Digit Span	
					
	n	Mean (SE)	Difference (SE)	Mean (SE)	Difference (SE)	Mean (SE)	Difference (SE)	Mean (SE)	Difference (SE)
Subclinical hyperthyroidism	30	7.64 (1.33)	-0.80 (1.33)	7.10 (1.30)	-1.43 (1.29)	8.36 (1.20)	-0.83 (1.18)	7.70 (1.15)	-0.79 (1.16)
Euthyroid	1275	8.44 (0.16)	0.00 (Reference)	8.52 (0.14)	0.00 (Reference)	9.19 (0.14)	0.00 (Reference)	8.49 (0.12)	0.00 (Reference)
Subclinical hypothyroidism	22	10.32 (1.40)	1.89 (1.44)	11.39 (0.75)	2.86** (0.78)	11.62 (0.72)	2.43** (0.73)	9.80 (0.69)	1.31 (0.69)

n, Sample size. SE, standard error. Difference, mean difference compared to the referent group. **, p < 0.01. Test for the mean difference was derived from the general linear model using SPSS Complex Sampling.

Table 3 shows the results from the general linear model adjusted for age, sex, ethnicity, poverty income ratio, test language, and examiner. Even after the adjustment, adolescents with subclinical hypothyroidism had significantly higher mean scores in reading and block design than euthyroid adolescents (the reference group).

**Table 3:** Adjusted differences¶ in mean cognitive assessment scores by thyroid status among adolescents: The Third National Health and Nutrition Examination Survey, 1988–94

		Arithmetic		Reading		Block Design		Digit Span	
					
	n	Mean Difference	SE	Mean Difference	SE	Mean Difference	SE	Mean Difference	SE
Subclinical hyperthyroidism	30	-0.05	1.14	-0.54	1.19	-0.12	0.95	-0.17	1.10
Subclinical hypothyroidism	22	1.14	1.22	2.20**	0.59	2.34**	0.77	0.95	0.65
Euthyroid	1702	0.00	Reference	0.00	Reference	0.00	Reference	0.00	Reference

¶Mean difference was derived from the general linear model with adjustment for age, sex, ethnicity/race, poverty income ratio, test language, and examiner using SPSS Complex Sampling.

n, Sample size. SE, Standard error. **, p < 0.05

## Discussion

This study of a national sample of adolescents in the United States found that adolescents with subclinical thyroid dysfunctions could have different cognitive performance. Our analysis found a higher average cognitive score in mild hypothyroidism and a lower average cognitive score in mild hyperthyroidism in adolescents in comparison to the euthyroid group. Mean scores in reading and block design were significantly higher in adolescents with subclinical hypothyroidism than the scores in the euthyroid group.

In children, contrary to congenital hypothyroidism, significant correlations between low thyroid hormone value and higher achievement in psychoeducational tests at diagnosis were observed in pediatric endocrinologists' practices. Behavior problems and decline in school performance after initiation of treatment for juvenile acquired hypothyroidism were recognized [15]. It was hypothesized that these children were overachievers in the thyroid-deficient state because of the decreased activity levels and lower arousal. We demonstrated that subclinical thyroid status was correlated with cognitive function. Cognitive performance tended to be better for adolescents with subclinical hypothyroidism. It is possible that these children were overachievers due to decreased activity levels and lower arousal also. Although our data does not support poor cognitive function in acquired juvenile subclinical hypothyroidism, it is possible that prolonged and severe forms of hypothyroidism might have negative effects on cognitive functions. Treatment of subclinical hypothyroidism in children with psychiatric disorders, behavior problems, and learning disabilities is not an uncommon practice. The benefit of such treatment is controversial. Our findings suggest that treatment decisions of subclinical hypothyroidism in children should be very cautious.

Mean scores of the cognitive assessments tended to be lower for adolescents with subclinical hyperthyroidism than that for adolescents with normal TSH levels, although the differences in the cognitive assessments did not reach statistical significance in this study. This may suggest that hyperthyroidism may be a potential risk factor affecting the cognitive performance among adolescents, though the effect could be indirect. Children with hyperthyroidism usually present with behavioral disturbances such as attention problems, difficulty in concentration, and hyperactivity, which may lead to poor cognitive performance [16]. Attention disturbances in children with hyperthyroidism become attenuated once euthyroidism is achieved through treatment [17].

Routine screening for thyroid disease with thyroid function tests is not recommended for asymptomatic children or adults [18]. The clinical impact of subclinical thyroid disease is not fully known. In this study, subclinical thyroid condition might be associated with cognitive performance; therefore monitoring thyroid function in adolescents with cognitive disorder could be helpful. The importance and timely diagnosis and treatment of subclinical thyroid disease, however, continue to be contentious research topics at this time.

This study used data from a national sample of adolescents residing in the United States, thus study findings could be applicable to the adolescents on a population-wide basis. However, the study has several limitations. First, the number of adolescents with T4 and TSH values beyond the reference ranges was rather small in this study. Thus, the study might have insufficient power to detect the differences between the groups in certain subscales of cognitive function. This might explain that compared to the euthyroid group, adolescents with subclinical hyperthyroidism appeared on average having a lower score in each of the cognitive subscales, but the difference did not reach statistical significance. We were also unable to perform meaningful analyses stratified by race/ethnicity or social economic status due to the limited sample size. Second, cognitive performance was assessed using the subsets of the Wide Range Achievement Test-Revised (WRAT-R) and the Wechsler Intelligence Scale for Children-Revised (WISC-R). The clinical significance of the differences in the cognitive assessments observed among adolescents may be limited by the lack of standards for the partial assessment scores. Furthermore, the NHANES III cognitive assessment scores were on average much lower than the standardized data for the subscales of WRAT-R and WISC-R even among the healthy adolescents. This has raised questions on the appropriateness of using NHANES III data to assess cognitive outcomes [19]. We realized that in NHANES III, the cognitive assessments were performed by trained technicians (not psychologists) and in a less controlled testing environment, which might not be comparable to the standard situation. However, the cognitive tests were applied to adolescents in NHANES III indifferently regarding their thyroid status. We believe that the assessment scores for the different groups would indicate the relative levels of cognitive function among the groups. The NHANES cognitive data has been used to examine the effects of environmental tobacco smoke, food insufficiency, and blood lead levels [20-22]. Therefore, our estimates on the relationships between the subclinical conditions and cognitive function were thus not likely distorted.

## Conclusion

Subclinical hypothyroidism was associated with better performance in some areas of cognitive functions while subclinical hyperthyroidism could be a potential risk factor.

## Abbreviations

NHANES III, Third National Health and Nutrition Examination Survey

T4, total thyroxine

TSH, thyroid stimulating hormone

WRAT-R, Wide Range Achievement Test-Revised

WISC-R, Wechsler Intelligence Scale for Children-Revised

## Competing interests

The author(s) declare that they have no competing interests.

## Authors' contributions

Dr. Tiejian Wu initiated the research idea and performed data analysis. All authors contributed to the research and analytical design. All authors involved in drafting the manuscript and have given final approval of the version to be published.

## Pre-publication history

The pre-publication history for this paper can be accessed here:


